# CDK9 inhibition constrains multiple oncogenic transcriptional and epigenetic pathways in prostate cancer

**DOI:** 10.1038/s41416-024-02810-8

**Published:** 2024-08-08

**Authors:** Razia Rahman, Muhammed H. Rahaman, Adrienne R. Hanson, Nicholas Choo, Jianling Xie, Scott L. Townley, Raj Shrestha, Ramin Hassankhani, Saiful Islam, Susanne Ramm, Kaylene J. Simpson, Gail P. Risbridger, Giles Best, Margaret M. Centenera, Steven P. Balk, Ganessan Kichenadasse, Renea A. Taylor, Lisa M. Butler, Wayne D. Tilley, Simon J. Conn, Mitchell G. Lawrence, Shudong Wang, Luke A. Selth

**Affiliations:** 1 Flinders University, College of Medicine and Public Health, Flinders Health and Medical Research Institute, Bedford Park, SA Australia; 2 Drug Discovery and Development, Clinical and Health Sciences, University of South Australia, Adelaide, SA Australia; 3 Biomedicine Discovery Institute Cancer Program, Prostate Cancer Research Group, Department of Anatomy and Developmental Biology, Monash University, Clayton, VIC Australia; 4 Flinders University, Freemasons Centre for Male Health and Wellbeing, Bedford Park, SA Australia; 5 Victorian Centre for Functional Genomics, Peter MacCallum Cancer Centre, Melbourne, VIC Australia; 6 The Sir Peter MacCallum Department of Oncology, University of Melbourne, Parkville, VIC Australia; 7 Department of Biochemistry and Pharmacology, University of Melbourne, Parkville, VIC Australia; 8 Peter MacCallum Cancer Centre, Melbourne, VIC Australia; 9 Cabrini Institute, Cabrini Health, Malvern, Melbourne, VIC Australia; 10 Melbourne Urological Research Alliance (MURAL), Monash Biomedicine Discovery Institute Cancer Program, Monash University, Clayton, VIC Australia; 11 South Australian Health and Medical Research Institute, Adelaide, SA Australia; 12 Faculty of Health and Medical Sciences, The University of Adelaide, Adelaide, SA Australia; 13 Beth Israel Deaconess Medical Center, Boston, MA USA; 14 Department of Medical Oncology, Flinders Medical Centre, Southern Adelaide Local Health Network, Adelaide, SA South Australia; 15 Biomedicine Discovery Institute Cancer Program, Department of Physiology, Monash University, Clayton, VIC Australia; 16 Dame Roma Mitchell Cancer Research Laboratories, Adelaide Medical School, The University of Adelaide, Adelaide, SA Australia

**Keywords:** Targeted therapies, Prostate cancer, Oncogenes, Drug development

## Abstract

**Background:**

Cyclin-dependent kinase 9 (CDK9) stimulates oncogenic transcriptional pathways in cancer and CDK9 inhibitors have emerged as promising therapeutic candidates.

**Methods:**

The activity of an orally bioavailable CDK9 inhibitor, CDKI-73, was evaluated in prostate cancer cell lines, a xenograft mouse model, and patient-derived tumor explants and organoids. Expression of CDK9 was evaluated in clinical specimens by mining public datasets and immunohistochemistry. Effects of CDKI-73 on prostate cancer cells were determined by cell-based assays, molecular profiling and transcriptomic/epigenomic approaches.

**Results:**

CDKI-73 inhibited proliferation and enhanced cell death in diverse in vitro and in vivo models of androgen receptor (AR)-driven and AR-independent models. Mechanistically, CDKI-73-mediated inhibition of RNA polymerase II serine 2 phosphorylation resulted in reduced expression of BCL-2 anti-apoptotic factors and transcriptional defects. Transcriptomic and epigenomic approaches revealed that CDKI-73 suppressed signaling pathways regulated by AR, MYC, and BRD4, key drivers of dysregulated transcription in prostate cancer, and reprogrammed cancer-associated super-enhancers. These latter findings prompted the evaluation of CDKI-73 with the BRD4 inhibitor AZD5153, a combination that was synergistic in patient-derived organoids and in vivo.

**Conclusion:**

Our work demonstrates that CDK9 inhibition disrupts multiple oncogenic pathways and positions CDKI-73 as a promising therapeutic agent for prostate cancer, particularly aggressive, therapy-resistant subtypes.

## Introduction

Prostate cancer is the most common non-cutaneous cancer in men and a major cause of cancer mortality [1]. Most prostate tumors are exquisitely dependent on androgens and the androgen receptor (AR) for growth. Therefore, the mainstay treatment for metastatic disease is androgen deprivation therapy (ADT), which suppresses androgen biosynthesis and/or binding of ligand to the AR (2). While most men respond to ADT, their cancer inevitably returns in an incurable and lethal form termed castration-resistant prostate cancer (CRPC) [2]. The majority of CRPC tumors retain AR expression/activity, supporting the utility of second-generation AR-targeted therapies such as enzalutamide, abiraterone, darolutamide, and apalutamide [3]. By contrast, a smaller subset of CRPC tumors lose dependence on the AR signaling axis and acquire aggressive variant states characterized by high cancer cell plasticity, which can manifest in neuroendocrine prostate cancer (NEPC) [4]. The high rates of mortality associated with both AR-driven and AR-independent CRPC underscores the need for new treatments.

The AR is a member of the steroid receptor subfamily of nuclear receptors that functions as an intracellular ligand-activated transcription factor to mediate androgen signaling actions. In prostate cancer cells, AR regulates a transcriptional program associated with growth, luminal differentiation, and survival. Mechanisms by which AR signaling is maintained or re-activated in CRPC despite continued ADT include *AR* gene amplification and over-expression, *AR* activating mutations, the emergence of constitutively active AR splice variants, altered expression of AR co-regulators and intracrine androgen synthesis [2]. Another mechanism that enhances AR in the castrate environment is alterations to its phosphorylation state [5]. AR phosphorylation by cyclin-dependent kinases (CDKs), such as AKT, MAP kinases, and Src, generally increases following ligand binding and plays a key role in regulating AR’s transcriptional and DNA-binding activity, turnover by the ubiquitin-proteasome system, and cellular localization, all of which converge to influence AR-driven growth [5]. One kinase regulator of AR is cyclin-dependent kinase 9 (CDK9), which phosphorylates AR at serine 81 (pSer81-AR), a modification that enhances AR’s transcriptional activity and prostate cancer cell growth [6, 7]. More recent work found that this post-translational modification enables androgen-independent activity of the AR [8] and that pSer81-AR is abundant in advanced disease states [9].

CDK9 also promotes several other key oncogenic signaling pathways in cancer. CDK9 bound to cyclin T forms the positive transcriptional elongation factor (P-TEFb), the primary function of which is to phosphorylate the RPB1 subunit of RNAPII at serine 2 (pSer2-RNAPII) to promote transcriptional elongation [10]. Genes encoding mRNAs and proteins with short half-lives are highly reliant on CDK9/P-TEFb whereas low-level (basal) gene transcription occurs independently of this enzyme, such that CDK9 activity has diverse effects on the transcription of individual genes [11–13]. The BCL-2 family of anti-apoptotic proteins, which includes BCL-2, MCL-1, and XIAP, are exquisitely dependent on CDK9 for their expression [14]. In addition to over-riding programmed cell death responses via upregulation of anti-apoptotic factors, CDK9 also promotes the expression and/or activity of oncogenic transcription factors such as MYC, NF-κB, BRD4, and STAT3, all of which are established drivers of prostate cancer progression [15–19]. Thus, targeting CDK9 in CRPC would be expected to disable multiple oncogenic drivers of growth and therapy resistance.

We previously described a novel orally bioavailable CDK9 inhibitor, CDKI-73, and provided preclinical evidence for its utility as a targeted therapeutic agent for leukemia, ovarian cancer, colorectal cancer, and melanoma [20–25]. Here, we evaluate CDKI-73’s activity in prostate cancer and demonstrate that it promotes apoptosis and inhibits signaling by AR, MYC, and BRD4. This results in potent anti-tumor activity in an array of prostate cancer models, including those representative of AR-independent disease states. Combining CDKI-73 with a BRD4 inhibitor exhibited impressive efficacy in aggressive CRPC organoid models of AR-driven and AR-independent disease. Collectively, our findings illustrate the potential of CDKI-73 as a novel therapy for lethal prostate cancer.

## Materials and Methods

### Analysis of CDK9 expression and copy number in published datasets

Data from The Cancer Genome Atlas cohort [26] were obtained from cBioPortal [27]. Data from the Beltran cohort [28] was obtained from dbGaP (accession number phs000909). To compare the TCGA and Beltran datasets, mRNA levels of *CDK9* were normalized to mRNA levels of *ACTB*. For Kaplan-Meier survival analysis of the TCGA cohort, median *CDK9* mRNA levels were used to stratify patients into low and high expression groups; recurrence events and time correspond to the “Disease Free Status” and “Disease Free (Months)” parameters. *CDK9* copy number data for the TCGA, Beltran, and Stand Up 2 Cancer [29] cohorts was obtained from cBioPortal.

### Cell culture

PC3, DU145, C4-2B, LNCaP, VCaP, and 22Rv1 human prostate carcinoma cells were obtained from the American Type Culture Collection. V16D and MR49F cells [30] were kindly provided by Prof Amina Zoubeidi. PC3 cells were maintained in RPMI-1640 containing 5% fetal bovine serum (FBS). DU145, C4-2B, LNCaP, and 22Rv1 cells were cultured in RPMI + 10% FBS. VCaP cells were maintained in Dulbecco’s Modified Eagle’s Medium containing 10% FBS, 1% sodium pyruvate, 1% MEM non–essential amino acids, and 0.1 nM 5α-dihydrotestosterone (DHT; Sigma). All cell lines were authenticated using short tandem repeat profiling in 2022 by CellBank Australia and underwent regular testing for mycoplasma contamination.

### Cell viability and growth assays

Cell viability assays using 3‐(4,5‐dimethylthiazol‐2‐yl)‐2,5‐diphenyltetrazolium bromide (MTT; Sigma‐Aldrich) were performed as described previously [24]. The concentrations of CDKI-73 required to reduce growth by 50% (GI_50_) were calculated using Graphpad Prism 7.02 (La Jolla, CA, USA). Trypan blue assays were performed as described previously [31]. We also assessed cell growth using an Incucyte S3 instrument (Sartorius, Göttingen, Germany). Briefly, LNCaP cells (7 × 10^3^ per well), 22Rv1 or PC3 cells (2.5 x 10^3^ per well) were plated in 96-well plates in a total volume of 100 µl RPMI media per well. After 24 h, 10 µl of either DMSO or CDKI-73 dissolved in media at the appropriate final concentrations were added. Plates were then imaged over a period of 96 or 120 h, collecting four images per well every 6 h with a 20x objective. The resultant images were analyzed for confluency using the Incucyte S3 software, adjusting the settings for image analysis using a small training set of images (~6) from DMSO control wells and low, intermediate and high doses of compound at end timepoints.

### Detection of apoptosis using Annexin V-PE/7-AAD

LNCaP cells (2 × 10^5^ per well) were seeded in 6-well plates and incubated at 37^o^C for 48 h before treatment. Treated cells were trypsinized followed by centrifugation at 400 *g* for 5 min. The collected cell pellets were double stained with Annexin V-PE (BD Biosciences) and 7-AAD (Invitrogen), as described previously [31]. Samples were analyzed using a CytoFLEX S flow cytometer (Beckman Coulter) within 45 min of staining. For each sample, 10,000 intact, single cells were counted and the data analyzed using CytExpert 2.1 software (Beckman Coulter). Annexin V/7-AAD negative cells were considered viable.

### Western blotting

Protein extraction from cells using RIPA buffer (10 mM Tris (pH 7.4), 150 mM NaCl, 1 mM EDTA, 1% Triton X-100, 1x Roche Complete protease inhibitor cocktail, 1x Thermo Halt phosphatase inhibitor cocktail) and Western blotting were performed as described previously [32]. Antibodies used for Western blotting were: RNAPII (C15200004, Diagenode; used at 1:2000); pSer2-RNAPII (C15200005-50, Diagenode; 1:2000); BCL-2 (15071S; Cell Signaling Technology; 1:500); MCL-1 (94296S, Cell Signaling Technology; 1:500); XIAP (2045S; Cell Signaling Technology; 1:500); AR (ab108341, Abcam; 1:1000); pSer81-AR (07-1375, Merck; 1:1000); c-MYC (9402S, Cell Signaling Technology; 1:500); and GAPDH (MAB374, Merck; 1:2500). Immunoreactive bands were visualized using Clarity Western ECL Substrate (Bio-Rad) or ECL Select (Biostrategy) in a ChemiDoc MP instrument (Bio-Rad).

### Quantitative real-time PCR analysis of mRNA expression

RNA was extracted from cells using miRNeasy Mini Kits (Qiagen), with on-column DNase treatment using RNase-free DNase Kits (Qiagen), according to the manufacturer’s instructions. Reverse transcription was carried out using iScript Reverse Transcriptase kits (Bio-Rad). qRT-PCR was performed using PowerTrack SYBR Green Supermix (Thermo Fisher Scientific) in triplicate, as described previously [33]. For normalization of qRT-PCR data, *GAPDH*, *HPRT1*, and *ACTB* were used (refer to figure legends for more detail). Primer sequences are shown in Supplementary Table 1.

### Immunohistochemistry

Immunohistochemical staining of formalin-fixed paraffin-embedded transurethral resection of the prostate (TURP) tissue sections (2-4 μm) was carried out essentially as described previously [34]. In brief, antigen retrieval was performed using citrate buffer, pH 6.5 (CDK9), or Tris-EDTA buffer, pH 9.0 (p63 and AMACR) in a Biocare Medical Nexgen decloaker at 115 °C for 15 min. CDK9 slides were incubated in avidin/biotin blocking kit (Invitrogen, 004303) for 5 min. All slides were then incubated at room temperature with 5% goat serum for 30 min. Primary antibodies were: anti-CDK9 (CST, C12F7; used at 1:200 overnight at 4 °C); anti-p63 (DAKO, Clone DAK-p63; used at 1:100 for 60 min at RT); anti-AMACR (Metagene, BIC-PPM225DSAA; used at 1:200 for 60 min at RT). Slides were then incubated at room temperature with goat anti-Rabbit (DAKO E0432 for AMACR and CDK9) or goat anti-Mouse (DAKO E0433 for p63) secondary antibodies at 1:400 for 60 min followed by horseradish peroxidase-conjugated streptavidin (DAKO, P0397) at 1:500 for 1 h. Positive cells were visualized with 3,30-diaminobenzidine (DAB; Sigma, D3939) or Ultraview Red (Metagene, BIC-PPM225DSAA) and counterstained with hematoxylin (Australian Biostain). Cancer cells in immunostained sections were distinguished from benign glands with the assistance of benign basal cell marker p63, and α-methylacyl CoA racemase (AMACR) staining, as described previously [35]. Quantitative image analysis for CDK9 staining intensity in benign and malignant glands was done using QuPath software (QuPath-0.4.3). Briefly, slides were scanned using a NanoZoomer digital slide scanner (Hamamatsu Photonics) and images were imported to QuPath from the NPD viewer (NPD.view2). After annotation of regions of interest, positive cell selection was performed using mean nuclear DAB optical density and three intensity threshold parameters to determine the percentage of low, medium, and high intensity CDK9-stained nuclei.

### RNA sequencing

LNCaP cells were seeded in 6-well plates and treated with CDKI-73 (250 nM) or vehicle (DMSO) (3 biological replicates of each). Total RNA was extracted using a PureLink™ RNA Mini Kit (Life Technologies, VIC, Australia). PolyA+ enriched RNA-seq libraries from biological replicates of the untreated and treated groups were multiplexed and sequenced on the Illumina NextSeq 500 platform at the Australian Cancer Research Foundation (ACRF) Cancer Genomics Facility using a stranded, single-end protocol with a read length of 75 bp. Raw data, averaging 73.2 million reads per sample, were analyzed and quality-checked using the FastQC program (http://www.bioinformatics.babraham.ac.uk/projects/fastqc). Reads were mapped against the human reference genome (hg38) using the STAR spliced alignment algorithm [36] (version 2.6.1d with default parameters and --chimSegmentMin 20, --quantMode GeneCounts), returning an average unique alignment rate of 86.7%. Differential expression analysis was evaluated from TMM normalized gene counts using R (version 3.2.3) and edgeR (version 3.3) [37], following protocols described previously [38]. Graphical representations of differentially expressed genes were generated using Glimma [39].

Gene Set Enrichment Analysis (Subramanian et al., 2005) was performed using the Broad Institute’s public GenePattern server; the input was normalized count data, which was analyzed with default parameters.

### Chromatin immunoprecipitation (ChIP)-sequencing

LNCaP cells (growing in 15 cm plates) were treated with 150 nM CDKI-73 or vehicle control for 48 h. Subsequently, cells were fixed with formaldehyde and ChIP was performed essentially as described previously [31] using 2 µg of a H3K27ac rabbit polyclonal antibody (Abcam ab4729). For each treatment condition, 2 biological replicates were generated. After DNA quantification with Qubit dsDNA HS assay (Thermo-Fisher Scientific), 5 ng of DNA (ChIP-enriched or input) was used for library preparation using a Qiaseq UltraLow Input Library Kit (Qiagen). Sequencing was performed on an Illumina Nextseq 500 platform (single-end protocol, 75 bp read length) at the South Australian Genomics Center (SAGC). Reads were mapped to the hg19 genome build using Bowtie2 [40]. Duplicate and non-uniquely mapping (MAPQ cutoff 20) reads were removed as described previously [41]. Diffbind (version 3.10.1, running in R version 4.3.0) was used to identify sites with differential H3K27ac intensity (*p* < 0.05) in response to CDKI-73 treatment. Overlaps between our data and other ChIP-seq datasets and peak sets were determined using Cistrome DB Toolkit [42] or BEDTools [43]. Deeptools [44] was used to convert BAM files to bigwigs and for visualizing ChIP-seq data. ChIPseeker (Galaxy Version 1.28.3) [45] was used to define genomic locations of H3K27ac peaks. HOMER [46] was used to identify known motifs enriched within peak sets (findMotifsGenome.pl -size 500).

### Animal studies

All protocols for animal experiments were approved by the Animal Ethics Committee of the University of South Australia, Adelaide, Australia (project number: U11-19) and all methods were carried out in accordance with their guidelines and regulations. Subcutaneous LNCaP xenografts were established as described previously [47]. Briefly, male BALB/c nude mice (nu/nu) aged 8 to 10 weeks were purchased from the Animal Resource Center (Canning Vale, WA, Australia) and housed at the University of South Australia’s Core Animal Facility in a pathogen-free environment with free access to food and water. Tumors were established on the right flanks of the mice by subcutaneous injection of 5 × 10^6^ LNCaP cells suspended in 1:1 growth factor-reduced Matrigel (In Vitro Technologies, VIC, Australia). Treatments were commenced after randomly allocating the mice into different groups when the average tumor volume reached ~150 mm^3^ (*n* = 9–10 mice per group). To calculate sample sizes, we used G*Power (version 3.1.9.6) [48] with an effect size of 0.7, a statistical power level of 0.8, and a significance level of 0.05. Tumor volume was calculated as described previously [49]. Before commencing treatments, mice were randomized into groups; the absence of a statistically significant difference in baseline tumor volume between groups was then confirmed using an independent sample *t*-test (*p* > 0.05). Investigators were blinded to group allocation but not when assessing experimental outcomes. CDKI-73 and AZD5153 were prepared in the vehicle (1% carboxymethyl cellulose) and administered orally (*per os*) at 50 mg/kg and 5 mg/kg, respectively; single agent treatments were administered on day 1 and then daily from day 3 whereas for the combination treatment drugs were administered on days 1, 3–7 and 10–11. Mice were humanely killed after 21 days of dosing or if they reached the clinical endpoint (tumor volume ≥1500 mm^3^ or ≥15% body weight loss). Tumors were collected in two separate aliquots and either fixed in 10% neutral buffered formalin or snap-frozen in liquid nitrogen for further molecular analyzes. No animals were excluded from the analyzes.

### Ex vivo culture of human prostate tumors

Human ethics approval was obtained from the University of Adelaide Human Ethics Committee (approval number H-2012-016) and all methods were carried out in accordance with their guidelines and regulations. Fresh prostate cancer specimens were obtained with written informed consent from men undergoing robotic radical prostatectomy at St Andrew’s Hospital, Adelaide, through the Australian Prostate Cancer BioResource. Tumor characteristics are summarized in Supplementary Table 2. Tissues were dissected and cultured as described previously [34] in a medium containing DMSO or CDKI-73. After 48 h of culture, tissues were formalin-fixed and paraffin embedded, sectioned, and stained for Ki67 and cleaved caspase-3 (CC-3) as described previously [50].

### Organoid culture and growth assays

Patient-derived xenografts (PDXs) generated by the Melbourne Urological Research Alliance (MURAL) have been described [51]. PDXs included tumors growing in non-castrate mice supplemented with a testosterone pellet or tumors growing in castrated mice, which have previously been designated using the suffix “Cx” [51]. Organoids were derived from patient-derived xenografts (PDXs) cultured as previously described [52] and used for manual growth assays and high-throughput growth assays. For the latter, organoids were embedded in Matrigel in 384-well plates and treated with compounds at the Victorian Center for Functional Genomics, as described previously [53]. For all experiments, organoid growth was measured using CellTiter-Glo Luminescent Cell Viability Assays (Promega) according to the manufacturer’s recommendations. The synergy between CDKI-73 and AZD5153 was determined using SynergyFinder Plus [54], a tool designed to analyze drug combination dose-response data. Average synergy scores from 3 major synergy scoring models, HSA, Bliss, and ZIP, were calculated; scores > 10 indicate that interaction between two drugs is likely to be synergistic, whereas scores between -10 and 10 indicate that interaction between two drugs is likely to be additive.

### Statistical analyzes

All statistical analyzes were carried out using GraphPad Prism (version 5; GraphPad Software, San Diego, CA, USA) or MedCalc (version 12; MedCalc Software, Mariakerke, Belgium). Details of statistical tests used are provided in the figure legends; all tests were two-sided. Evaluation of normality was done using Kolmogorov-Smirnov tests when sample sizes were ≥5; for experiments with smaller sample sizes, normality was assumed.

## Results

### CDK9 is over-expressed in prostate cancer

**Fig. 1:**
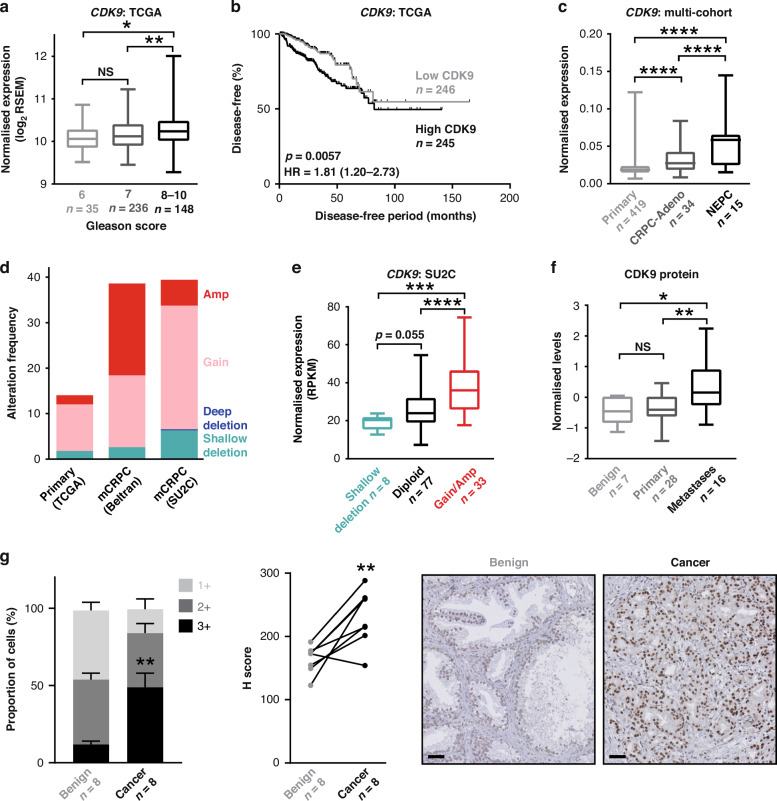
CDK9 is commonly over-expressed and amplified in prostate cancer. **a** Expression level of *CDK9* in localized prostate cancer according to increasing Gleason score in the TCGA [26] cohort. Boxes extend from the 25th to 75th percentiles; the middle line is the median; top and bottom lines are minimum and maximum. Groups were compared using ANOVA and Tukey’s multiple comparison tests. **b** Kaplan–Meier curve showing estimated disease-free probability following radical prostatectomy in patients with high or low levels of *CDK9* in the TCGA cohort. P value and hazard ratio were determined using log-rank tests. **c** Expression level of *CDK9* in localized prostate cancer (TCGA) and adenocarcinoma (CRPC-Adeno) or neuroendocrine (NEPC) subtypes of CRPC [28]. *CDK9* mRNA levels were normalized to *ACTB* mRNA levels. Groups were compared using ANOVA and Tukey’s multiple comparison tests. **d** *CDK9* is commonly amplified in prostate cancer. Data is from The Cancer Genome Atlas (TCGA), Beltran [28] and Stand Up 2 Cancer (SU2C) [29] cohorts. **e** *CDK9* gene amplification in the SU2C cohort is associated with increased mRNA expression. Groups were compared using ANOVA and Tukey’s multiple comparison tests. **f** CDK9 protein expression in clinical prostate samples (benign, primary tumors, or bone metastases) was measured by mass spectrometry [55]. Groups were compared using ANOVA and Tukey’s multiple comparison tests. **g** CDK9 protein expression is elevated in malignant compared to benign prostate tissues. Left, stacked bar graph showing the proportion of cells with low (1+), intermediate (2+), and high (3+) intensity CDK9 staining. Error bars are standard errors of the mean (s.e.m.). *P* value was determined using an unpaired t-test comparing the 3+ proportions. Middle, H scores of matched tumors and non-malignant samples (*n* = 8). P value was determined using a paired t test. Right, representative staining of non-malignant and Gleason grade 3 and 4 tumors (bar = 100 µm). In all panels, *p* values are: *p < 0.05; **p < 0.01; ***p < 0.001; ****p < 0.0001.

To garner evidence for CDK9 as a therapeutic target in prostate cancer, we assessed its expression in clinical transcriptomic datasets. In silico analyzes of The Cancer Genome Atlas (TCGA; [26]) cohort indicated that *CDK9* expression is elevated in high Gleason score primary tumors (Fig. 1a) and associated with disease recurrence (Fig. 1b). Moreover, *CDK9* mRNA levels are higher in CRPC compared to primary tumors (Fig. 1c). Interestingly, *CDK9* mRNA levels were significantly higher in castration-resistant neuroendocrine prostate cancer (NEPC) tumors, which exhibit AR-low/null/independent phenotypes, compared to the more common CRPC adenocarcinoma phenotype (Fig. 1c). Gain or amplification of the *CDK9* gene is common in prostate cancer, particularly in CRPC (Fig. 1d), and copy number alterations explain, at least in part, increased *CDK9* mRNA expression (Fig. 1e). CDK9’s association with metastatic disease was recapitulated at the protein level in patient samples in which proteomes were profiled using mass spectrometry [55] (Fig. 1f). We further examined CDK9 protein expression by immunohistochemistry (IHC) in castration-sensitive prostate tumors, obtained via transurethral resection of the prostate. CDK9 was expressed in almost all prostate epithelial cells, localized to the nucleus, and was more abundant in cancer compared to non-malignant samples (Fig. 1g). In summary, CDK9 is highly expressed in prostate cancer, particularly tumors with aggressive phenotypes, reinforcing its potential relevance as a therapeutic target.

### CDKI-73 promotes apoptosis and inhibits CDK9 activity in prostate cancer cells

**Fig. 2:**
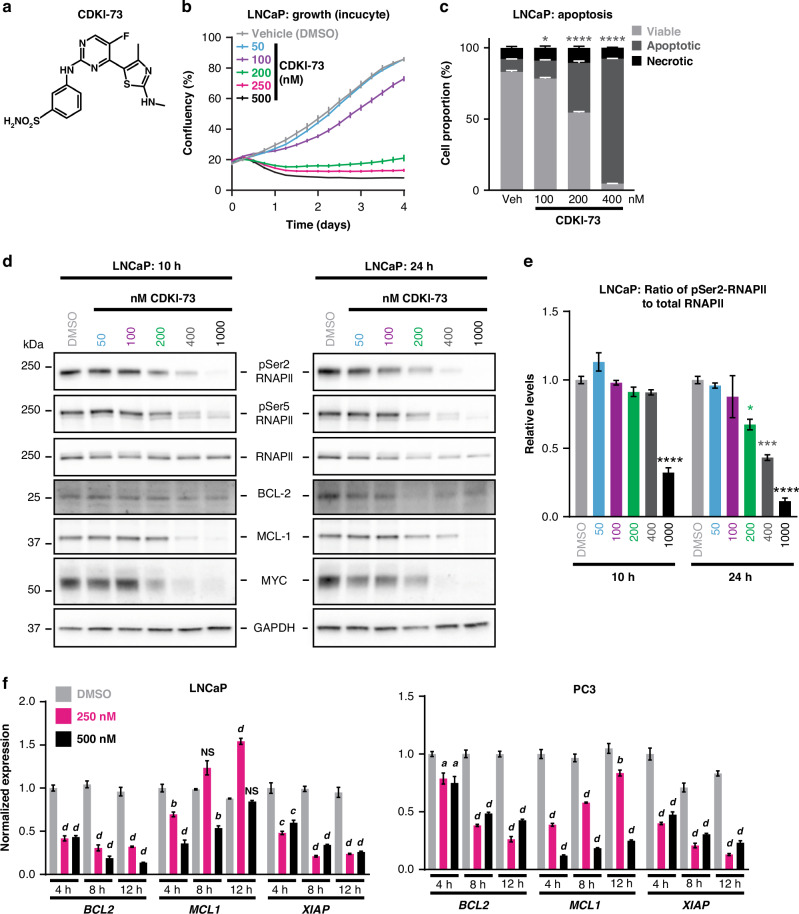
CDKI-73 causes cell death and suppresses anti-apoptotic pathways in prostate cancer cells. **a** Chemical structure of CDKI-73. **b** Live-cell confluency analysis (Incucyte) demonstrates a dose-dependent reduction of LNCaP cell growth in response to CDKI-73. Error bars are ± s.e.m. of 6 biological replicates. Data is representative of 3 independent experiments. **c** Flow cytometry-based Annexin V/7-AAD apoptosis assay after 72 h of treatment with the indicated doses of CDKI-73. Data represents the mean of triplicate samples and are representative of 3 independent experiments. Error bars are s.e.m. Apoptotic cell proportions were compared to the vehicle using ANOVA and Tukey’s multiple comparison tests (*p < 0.05; ****p < 0.0001). **d** Representative Western blots showing decreased levels of RNAPII, pSer2-RNAPII, BCL-2, MCL-1, and MYC following treatment of LNCaP cells with the indicated doses of CDKI-73 or vehicle control (DMSO) for 10 or 24 h. GAPDH is shown as a loading control. **e** Levels of pSer2-RNAPII normalized to total RNAPII (both normalized to GAPDH) following treatment of LNCaP cells with the indicated doses of CDKI-73 or vehicle control (DMSO) for 10 or 24 h. This data is from an independent experiment to that shown in panel **d**. **f** Expression of genes encoding anti-apoptotic factors, as measured by qRT-PCR, following 4, 8, or 12 h of treatment with the indicated doses of CDKI-73 or vehicle control (DMSO). Gene expression was normalized to *GAPDH*, *HPRT1*, and *ACTB*; expression for DMSO (4 h) was set to 1. Error bars are ± s.e.m. of 3 biological replicates; P values (treatment compared to vehicle) were determined using ANOVA and Dunnett’s multiple comparisons tests. The data shown is representative of 3 independent experiments. In all panels: *a* or *p < 0.05; *b* or **p < 0.01; *c* or ***p < 0.001; *d* or ****p < 0.0001; NS, not significant.

CDKI-73 is a heterocyclic 3-(5-fluoro-4-(4-methyl-2-(methylamino)thiazol-5-yl)pyrimidin-2-ylamino)benzenesulfonamide (Fig. 2a) that was rationally designed and optimized using a structure-guided approach [56]. The effect of CDKI-73 on prostate cancer cell viability was first assessed in a panel of cell lines using MTT assays. CDKI-73 exhibited potent activity against all models tested, with half-maximal growth inhibitory concentration (GI_50_) values ranging from 40–76 nM following 72 h of exposure (Supplementary Fig. 1a, b). Two non-malignant lung fibroblast cell lines, MRC-5 and WI-38, were markedly less sensitive to CDKI-73 (GI_50_ values of 7.4 and 2.2 µM, respectively) than the prostate cancer cell lines (Supplementary Fig. 1a, b); however, it must be noted that the lung fibroblast models represent an alternative cell lineage, which may explain the discrepancy in responses to CDKI-73. The anti-proliferative activity of CDKI-73 in PC3, LNCaP and 22Rv1 cells was validated using live growth analysis in an Incucyte instrument (Fig. 2b; Supplementary Fig. 1c). Flow cytometric analysis of annexin-V and 7-AAD revealed that CDKI-73 caused apoptosis of prostate cancer cells (Fig. 2c).

To confirm that the activity of CDKI-73 was associated with inhibition of CDK9 activity, we first analyzed relevant protein readouts by Western blotting. Treatment of LNCaP cells with CDKI-73 for 10 or 24 h reduced the levels of pSer2-RNAPII, even accounting for a minor reduction in total RNAPII that was also observed (Fig. 2d, e). Since CDKI-73 can also inhibit CDK7 [20, 21], we measured RNAPII phosphorylation at serine 5 (pSer5-RNAPII), a key substrate of this kinase; as expected, pSer5-RNAPII was also decreased in response to treatment (Fig. 2d). Concomitant with its effects on RNAPII phosphorylation, CDKI-73 treatment also led to a robust decrease in the levels of MYC and the pro-survival proteins BCL-2 and MCL-1 (Fig. 2d). Consistent with these observations, CDKI-73 treatment of LNCaP caused a rapid down-regulation of BCL-2 family members *BCL2*, *MCL1* and *XIAP* mRNA (Fig. 2f). CDKI-73-mediated loss of pSer2-RNAPII, anti-apoptotic proteins and MYC was confirmed in two CRPC cell line models, 22Rv1 and PC3 (Fig. 2f; Supplementary Fig. 2). Collectively, these pharmacodynamic analyzes confirm that CDKI-73 can inhibit the activity of CDK9 in prostate cancer cells.

### CDKI-73 has potent anti-tumor activity in diverse, clinically-relevant models of aggressive prostate cancer

**Fig. 3:**
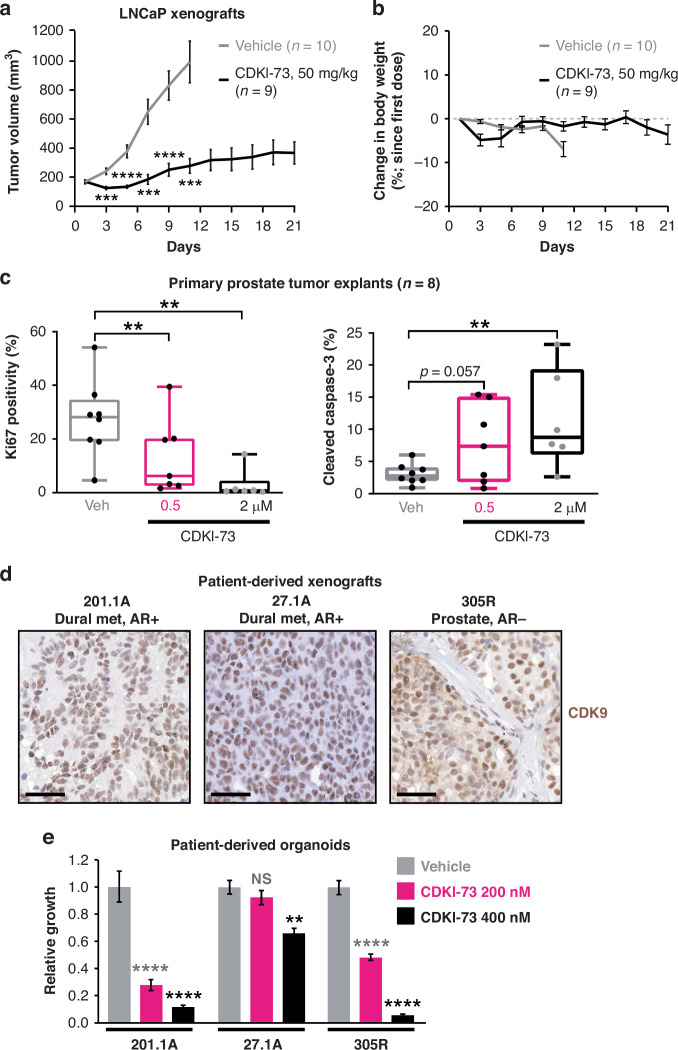
CDKI-73 has potent anti-tumor activity in diverse, clinically-relevant models of aggressive prostate cancer. **a** Growth of LNCaP xenografts in mice treated with vehicle (*n* = 10) or CDKI-73 (*n* = 9). CDKI-73 (50 mg/kg) was administered orally on day 1 and then daily from day 3. For vehicle-treated mice, tumor size in 4 mice reached the ethical end-point at day 11; hence, this is the final time-point in this group. Graphs show the mean ± s.e.m. at each time-point. **b** Average body weight of mice harboring LNCaP xenografts and treated with vehicle or CDKI-73 (50 mg/kg). For vehicle-treated mice, tumor size in 4 mice reached the ethical end-point at day 11; hence, this is the final time-point in this group. **c** CDKI-73 inhibits proliferation and promotes cell death in prospectively collected human tumors grown as patient-derived explants (PDEs). PDEs (from *n* = 8 patients) were treated for 48 h. Ki67 and cleaved caspase-3 were evaluated by immunohistochemistry. Boxes extend from the 25th to 75th percentiles; the middle line is the median; top and bottom lines are minimum and maximum. Groups were compared using ANOVA and Tukey’s multiple comparison tests. **d** Representative immunohistochemistry staining of CDK9 in 3 independent PDXs, 27.1A, 201.1A and 305R. Source of tissue for the PDXs and AR status are shown in Supplementary Fig. 3. Met, metastasis. The bar is 50 µM. **e** CDKI-73 inhibits the growth of organoids grown from PDXs. Organoid viability was determined using Cell Titer Glo viability assays 3 days post-treatment. Data represents the mean ± s.e.m. of quadruplicate samples; treatment groups were compared to vehicles using ANOVA and Tukey’s multiple comparison tests. In all panels: *p < 0.05; **p < 0.01; ***p < 0.001; ****p < 0.0001; NS, not significant.

Encouraged by the promising activity of CDKI-73 in prostate cancer cell lines, we turned to more clinically relevant models of disease. First, we evaluated the in vivo efficacy of CDKI-73 in LNCaP xenografts, a widely used AR-driven, hormone-sensitive model of disease. Mice harboring subcutaneous LNCaP xenografts were treated orally with CDKI-73 (50 mg/kg) or vehicle daily for 21 days, a protocol based on previous dosage regimens for this compound [22, 24]. Treatment with CDKI-73 caused a significant reduction in tumor volume, estimated to be ~72% reduction at day 11, at which point mice in the vehicle group were humanely killed because tumor sizes had reached the ethical endpoint (Fig. 3a). Throughout the CDKI-73 treatment period, there was no significant change in animal body weight (Fig. 3b) or other overt signs of clinical toxicity.

Additional preclinical studies were performed using a system that allows for ex vivo culture of patient-derived primary tumors obtained from prostatectomy [50, 57]. Tissues obtained from 8 patients with intermediate-grade tumors (Supplementary Table 2) were cultured as explants with 0.5 or 2 µM CDKI-73 for 48 h, after which fixed tissues were stained for Ki67 and cleaved caspase-3 to assess effects on proliferation and apoptosis, respectively. In these clinical specimens, CDKI-73 elicited a significant and dose-dependent decrease in tumor cell proliferation (~90% decrease with 2 µM CDKI-73) and an increase in apoptosis (~375% increase with 2 µM CDKI-73) (Fig. 3c).

Finally, we utilized patient-derived models that are representative of aggressive prostate cancer [51, 58] (Supplementary Fig. 3). Patient-derived xenografts (PDXs) 27.1A and 201.1A were derived from brain and dural metastases, respectively, obtained from CRPC patients treated with various first- and second-generation AR-targeted therapies and chemotherapies [58]; both are PSA-positive and exhibit alterations to the *AR* gene that mediate therapy resistance [58] (Supplementary Fig. 3). PDX 305R was derived from a radical prostatectomy specimen obtained from a patient who died rapidly from their disease; it is a de novo large cell neuroendocrine tumor negative for AR and expressing markers of neuroendocrine differentiation [51] (Supplementary Fig. 3). Tissues obtained from the 27.1A, 201.1A and 305R PDXs were all strongly positive for CDK9 (Fig. 3d). When the PDXs were grown as organoid cultures [52], CDKI-73 potently inhibited the growth and viability of 201.1A and 305R and had a less pronounced, but still significant, effect on 27.1A (Fig. 3e). Collectively, our work demonstrates that CDKI-73 has activity in a diverse array of aggressive prostate cancer subtypes, including AR-driven and AR-independent disease.

### CDKI-73 perturbs androgen receptor signaling in prostate cancer cells

**Fig. 4:**
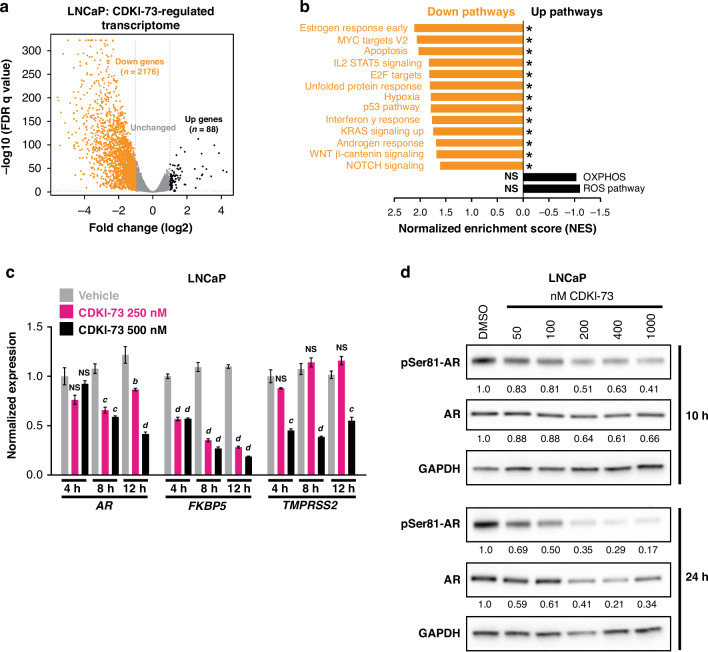
CDKI-73 reduces AR expression and activity. **a** Volcano plot demonstrating genes differentially expressed (≥2-fold change and false-discovery rate (FDR) *q* value ≤ 0.05) in response to 4 h of treatment with 250 nM CDKI-73. **b** Hallmark pathways [75] suppressed (down) and activated (up) by CDKI-73, as determined using GSEA. NS, not significant. **c** Expression of *AR* mRNA and AR target genes (*FKPB5* and *TMPRSS2*), as measured by qRT-PCR, following 4, 8 or 12 h of treatment with the indicated doses of CDKI-73 or vehicle control. Gene expression was normalized to *GAPDH*, *HPRT1*, and *ACTB*; expression for the vehicle (4 h) was set to 1. Error bars are ± s.e.m. of 3 biological replicates; *P* values (treatment compared to vehicle) were determined using ANOVA and Dunnett’s multiple comparisons tests. The data shown is representative of 3 independent experiments. **d** Representative Western blots showing levels of pSer81-AR and total AR following treatment of LNCaP cells with the indicated doses of CDKI-73 or vehicle control (DMSO) for 10 and 24 h. GAPDH is shown as a loading control. The values below the pSer81-AR blots are levels of pSer81-AR normalized to total AR (both normalized to GAPDH, with DMSO set to 1); the values below the AR blots are its levels normalized to GAPDH (with DMSO set to 1). In all panels: *a* or *p < 0.05; *b* or **p < 0.01; *c* or ***p < 0.001; *d* or ****p < 0.0001; NS, not significant.

To measure the global effects of CDK9 inhibition on transcription in prostate cancer, we undertook RNA sequencing in LNCaP cells treated with CDKI-73. The treatment period was 4 h, a time point chosen to interrogate acute alterations to the transcriptome. CDKI-73 had a profound impact on transcription, modulating the expression of 2264 genes (Fig. 4a). The vast majority of altered genes were downregulated (~96%; 2176 downregulated versus 88 upregulated genes, Fig. 4a), as expected given CDKI-73’s mode of action. Recapitulating our earlier targeted experiments, levels of genes encoding anti-apoptotic factors and *MYC* were significantly decreased by CDKI-73 (Supplementary Fig. 4a). Supporting these gene-level observations, pathway-level analyzes demonstrated that downregulated genes were enriched in the hallmark gene sets of apoptosis, E2F targets and MYC targets (Fig. 4b). Moreover, CDKI-73 caused suppression of a 31-gene cell cycle progression (CCP) signature that has been validated for its capacity to predict progression to metastatic disease and death from prostate cancer [59, 60] (Supplementary Fig. 4b).

The transcriptomic data also revealed significant suppression of the androgen response pathway by CDKI-73 (Fig. 4b) and we confirmed down-regulation of core AR target genes using qRT-PCR (Fig. 4c; ~2-fold reduction *TMPRSS2* mRNA and ~4-fold reduction of *FKBP5* mRNA at 12 h). *AR* mRNA was also decreased in response to CDKI-73 but this occurred later than its target genes (Fig. 4c), leading us to speculate that reduced AR target gene expression may also be a consequence of defective phosphorylation of Ser81-AR by CDK9 [6–8]. To investigate this in more detail, we measured total AR and pSer81-AR after treatment of LNCaP cells with CDKI-73. Cells were grown in standard culture media (i.e. RPMI-1640 containing 10% FBS), which contains sufficient levels of androgen to stimulate AR activity. Within 10 h, pSer81-AR was reduced by CDKI-73 treatment in a dose-dependent manner with an accompanying reduction, albeit to a lesser extent, in total AR (Fig. 4d). At a later time-point (24 h), the CDK9 inhibitor caused a substantial loss of pSer81-AR and total AR (Fig. 4d; ~70–80% reduction with 400 nM dose). Collectively, these data suggest that one mode of action of CDKI-73 is to reduce AR expression and activity in prostate cancer cells.

To confirm this finding, we utilized a distinct AR-positive prostate cancer cell line, 22Rv1, which is commonly used to model CRPC. Recapitulating the results from LNCaP cells, treatment of 22Rv1 cells with CDKI-73 for 10 or 24 h decreased AR pSer-81 levels (Supplementary Fig. 5; ~55% reduction in AR pSer-81 with 500 nM CDKI-73 at either time-point). Total AR levels were also reduced by CDKI-73 (Supplementary Fig. 5), recapitulating what was observed in LNCaP cells. Due to an *AR* gene rearrangement [61], 22Rv1 cells express high levels of C-terminally truncated AR variants (AR-Vs), most notably AR-V7, which are constitutively active (i.e. androgen-independent) and can mediate resistance to AR-targeted therapies [62]. Importantly, CDKI-73 caused a substantial loss of serine 81 phosphorylated AR-Vs (~80% reduction with 500 nM CDKI-73 at 24 h) and also reduced total levels of the truncated AR isoforms (Supplementary Fig. 5). These results provide evidence that CDKI-73 can inhibit the activity of both full-length and truncated AR variants in advanced prostate cancer.

### CDKI-73 induces epigenomic reprogramming and disrupts prostate cancer-associated enhancers

**Fig. 5:**
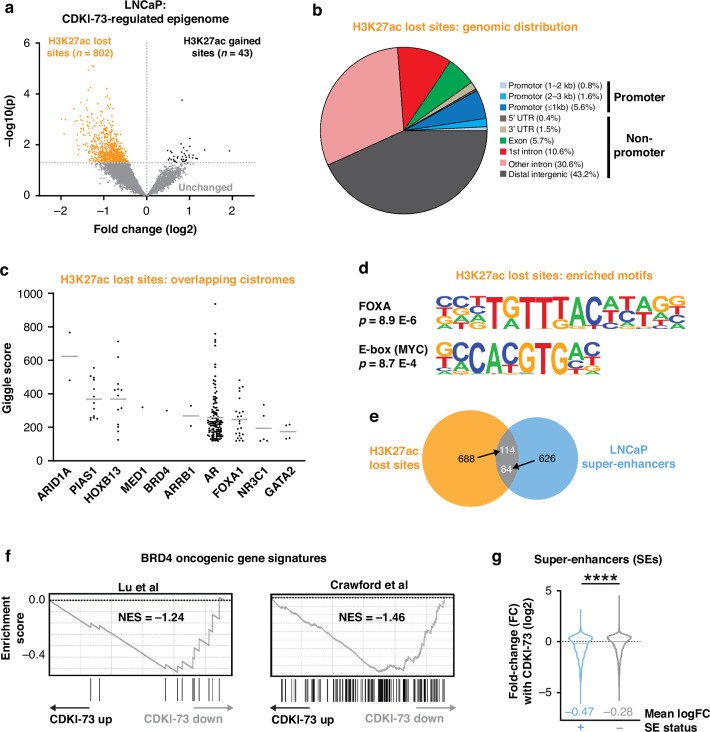
CDKI-73 induces epigenomic reprogramming and disrupts prostate cancer-associated enhancers. **a** Volcano plot demonstrating genomic sites with differential H3K27ac ChIP-seq signal in response to 48 h of treatment with 150 nM CDKI-73. **b** Genomic distribution of H3K27ac lost sites, determined using ChIPseeker [45]. **c** GIGGLE plot showing overlap between H3K27ac lost sites and publicly available cistrome data for transcription factors and epigenetic regulators. Points indicate individual datasets, line indicates mean score. **d** Motifs enriched in the H3K27ac lost sites, were identified using HOMER [46]. *P* values represent enrichment over the genomic background. **e** Overlap between H3K27ac lost sites and LNCaP super-enhancers defined in a previous study [76]. Arrows indicate the number of sites in that particular dataset that overlap with the other. **f** CDKI-73 suppresses BRD4 oncogenic gene sets [68, 77], as demonstrated by GSEA. NES, normalized enrichment score. **g** Fold-change of mRNAs following CDKI-73 treatment for genes associated with or without super-enhancers (SE status + and -, respectively). LNCaP genes with SEs were obtained from the SEdb 2.0 database [78] using published H3K27ac ChIP-seq data (GSM1902615) [76].

The transcriptomic profiling revealed acute changes to gene expression in response to CDKI-73. Since it impinges on the activity of key transcriptional regulators, including AR and MYC, we hypothesized that CDKI-73 would also reprogram the epigenome subsequent to these acute transcriptional effects. To address this hypothesis, ChIP-seq was used to profile acetylation of histone H3 lysine 27 (H3K27ac), a histone modification that is enriched at enhancers and promoters of actively transcribed genes. Following 48 h of treatment with CDKI-73, no substantial change in overall H3K27ac signal was observed at promoters or enhancers (Supplementary Fig. 6). However, using DiffBind [63], we identified 845 sites with altered H3K27ac signal in response to CDKI-73, 802 (95%) of which exhibited loss of this histone modification (“H3K27ac lost”, Fig. 5a; Supplementary Dataset 1). The vast majority (~92%) of the differential sites were in enhancers, with only ~8% being observed within 3 kb of transcriptional start sites (Fig. 5b). GIGGLE analysis revealed a significant overlap between H3K27ac lost sites and the cistromes of key transcriptional and epigenetic regulators of prostate cancer, including AR, FOXA1 and HOXB13 (Fig. 5c), supporting their functional relevance. This concept was further strengthened by motif analysis, which demonstrated enrichment of FOXA and MYC motifs within H3K27ac lost sites (Fig. 5d). We expanded our analysis to super-enhancers (SEs), cis-regulatory elements with high enrichment of H3K27ac that play a key role in regulating prostate cancer-related transcriptional programs [64], and found that 114 (~14%) of the H3K27ac lost sites overlapped with LNCaP SEs (Fig. 5e). Overall, these H3K27ac epigenomic analyzes demonstrate that CDKI-73 causes selective changes to H3K27ac, most notably loss of this activating mark at prostate cancer-associated enhancers.

### CDKI-73 synergizes with a BRD4 inhibitor

Prostate cancer-associated SEs are frequently bound and activated by bromodomain-containing protein 4 (BRD4) [65], a transcriptional and epigenetic regulator with a prominent function in prostate cancer progression [66]. Indeed, we noted that H3K27ac-lost sites were enriched for BRD4 binding events (Fig. 5c). Moreover, our RNA-seq data provided evidence for reduced BRD4 activity in response to CDKI-73 treatment (Fig. 5f) and also showed that SE-associated genes were more strongly down-regulated by CDKI-73 compared to non-SE genes (Fig. 5g). The ability of CDKI-73 to disrupt BRD4 function aligns with reports that CDK9-mediated phosphorylation of BRD4 enhance its activity [19]. Interestingly, elevated CDK9 activity is a mechanism of resistance to BET bromodomain inhibitors [67], and vice versa [50].

**Fig. 6:**
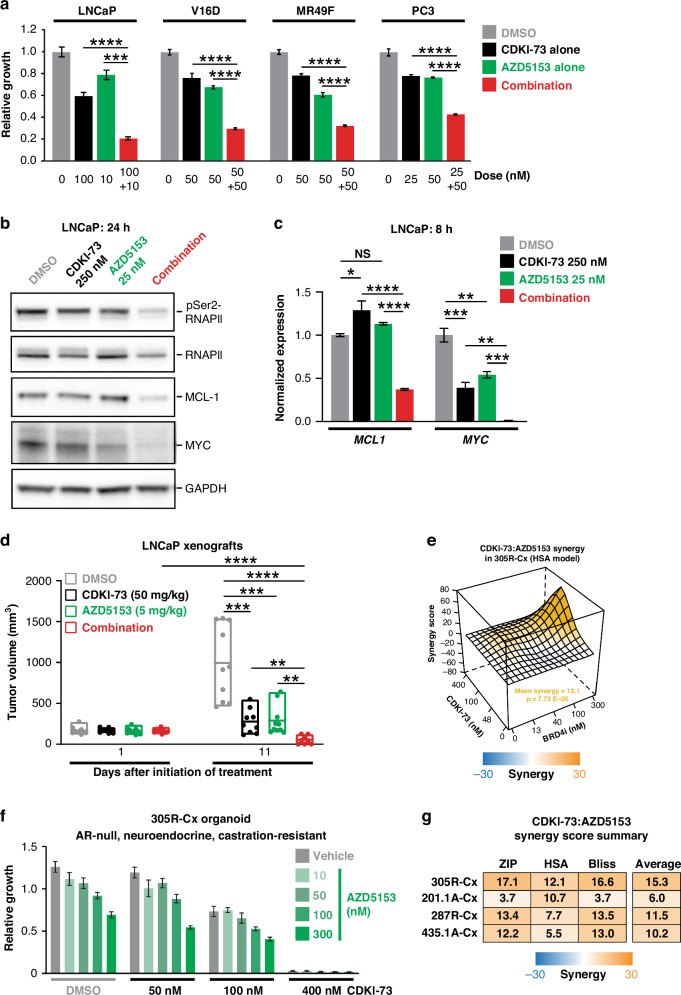
Synergism of CDKI-73 with BRD4 inhibition. **a** Growth assays assessing the effect of CDKI-73, AZD5153, or the combination of both drugs in the indicated cell lines. Error bars are ± s.e.m. Treatment groups were compared using t-tests. **b** Representative Western blots showing levels of pSer2-RNAPII, MCL-1, and MYC in LNCaP cells following 24 h of treatment with CDKI-73, AZD5153, or the combination. GAPDH is shown as a loading control. **c** Expression of *MCL1* and *MYC* mRNA, as measured by qRT-PCR, in LNCaP cells following 8 h of treatment with the indicated doses of CDKI-73, AZD5153 or the combination. Gene expression was normalized to *GAPDH* and *ACTB*; expression for vehicle (DMSO) was set to 1. Error bars are ± s.e.m. of 3 biological replicates; P values (treatment compared to vehicle) were determined using ANOVA and Tukey’s multiple comparisons tests. The data shown is representative of 2 independent experiments. **d** LNCaP tumor volume in mice treated with vehicle (*n* = 10), CDKI-73 (*n* = 9), AZD5153 (*n* = 10), or the combination (*n* = 9) following 1 or 11 days of engraftment. Single-agent treatments were administered orally on day 1 and then daily from day 3; for the combination treatment, drugs were administered orally on days 1, 3–7, and 10–11. Boxes are minimum to maximum; the line indicates the mean. Groups were compared using ANOVA and Tukey’s multiple comparison tests. **e** Synergistic activity of CDKI-73 and AZD5153 in 201.1A-Cx organoids. Organoid viability was determined using Cell Titer-Glo viability assays at 3 days post-treatment. Data represents the mean ± s.e.m. of 6 individual wells. **f** Synergy map (highest single agent (HSA) model) for the experiment shown in **e**, generated using SynergyFinder Plus [54]. **g** Summary of synergy between CDKI-73 and AZD5153 in 4 organoid models of aggressive prostate cancer. Mean synergy scores from 3 distinct synergy models (zero interaction potency (ZIP), HSA, and Bliss) are shown; the average of the 3 models is shown on the right. In all panels: *p < 0.05; **p < 0.01; ***p < 0.001; ****p < 0.0001; NS, not significant.

The interconnected functions of CDK9 and BRD4 have led to the evaluation of combinatorial targeting of both factors as a cancer therapy [67–69], a concept we explored in prostate cancer. More specifically, we assessed the combined efficacy of CDKI-73 and AZD5153, a small molecule inhibitor of BRD4 that is being tested in clinical trials as a therapy for advanced blood and solid cancers (e.g. NCT03205176). In 4 prostate cancer cell lines, which collectively model castration-sensitive disease (LNCaP), AR-positive CRPC (V16D), AR-positive enzalutamide-resistant CRPC (MR49F) and AR-negative CRPC (PC3), we observed a significant additive anti-proliferative effect when combing CDKI-73 and AZD5153 (Fig. 6a). The combination of the two drugs also elicited more pronounced effects on the protein and mRNA levels of pSer2-RNAPII, MCL-1 and MYC (Fig. 6b, c). This finding was recapitulated in vivo, with the combination therapy causing regression of established subcutaneous LNCaP xenografts and exhibiting significantly increased potency compared to the single agents (Fig. 6d).

Further evaluation of the CDKI-73/AZD5153 combination therapy was undertaken in the PDX-derived organoid models (Supplementary Fig. 3). More specifically, we employed a protocol in which organoids are robotically embedded in Matrigel in 384-well plates, permitting high-throughput drug screening [53]. In this experimental set-up, single-agent CDKI-73 exhibited similar anti-growth activity (Fig. 6e) to our experiments in which the organoids were seeded manually in 96-well plates (Fig. 3e). This high-throughput approach revealed synergy between CDKI-73 and AZD5153 in 305R-Cx (AR-negative, neuroendocrine) organoids and an additive effect of these drugs in 201.1A-Cx (AR-positive, CRPC-adenocarcinoma) organoids (Fig. 6e–g; Supplementary Fig. 7), determined using the SynergyFinder Plus tool [54] (see Materials and Methods). We expanded the work to 2 other models, 287R, and 435.1A (Supplementary Fig. 3): 287R-Cx was derived from a castration-sensitive tumor obtained via radical prostatectomy and is positive for AR, PSA, PSMA, and ERG but negative for neuroendocrine markers, whereas the 435.1A-Cx model was derived from a CRPC brain metastasis and is representative of AR-negative NEPC [51]. Strengthening earlier findings, a synergistic anti-growth effect of CDKI-73 and AZD5153 was also observed in 287R-Cx and 435.1A-Cx (Fig. 6g; Supplementary Fig. 7). Collectively, these data reveal the potential of combining CDKI-73 with a BRD4 inhibitor as a novel treatment for aggressive prostate cancer, including both AR-driven and AR-independent disease states.

## Discussion

CRPC is incurable and a major cause of cancer-related death in men; hence, new treatment strategies are urgently required. In this study, we highlight the relevance of CDK9 in promoting the growth and survival of aggressive prostate cancer and reveal the potential therapeutic utility of a CDK9 inhibitor, CDKI-73.

Mammalian CDKs can be broadly divided into two major classes: the first (CDK1, 2, 4, and 6) possess specialized functions in cell cycle control, whereas the second (CDK7–9, 12, 13, and 19) are critical for transcriptional regulation. CDK9 and CDK7 phosphorylate the carboxy-terminal domain (CTD) of RNAPII to regulate its activity; CDK7 promotes the initiation of transcription, whereas CDK9 mediates a switch to transcription elongation. Since elevated transcriptional rate is a hallmark of many tumors, there is strong interest in targeting CDK9 to treat cancer [10, 70]. Our evaluation of CDK9 expression in clinical prostate tissues supports this concept in a prostate cancer context: more specifically, we found that CDK9 expression is associated with tumor grade and is predictive of disease recurrence following surgery. Importantly, CDK9 expression is highest in CRPC, including AR-low/independent NEPC tumors. Mechanistically, this can be explained at least in part by the increased copy number of the *CDK9* gene, which is particularly evident in NEPC.

Given the emerging evidence for CDK9’s cancer-promoting activity [71] and its over-expression in prostate cancer, we investigated the therapeutic utility of an orally deliverable CDK9 inhibitor, CDKI-73. We have previously demonstrated anti-tumor efficacy of CDKI-73 in preclinical studies of acute myeloid leukemia [22], ovarian cancer [20, 23], colorectal cancer [24], and melanoma [25]. The findings herein demonstrate that this drug exerts anti-proliferative and pro-apoptotic effects in an array of prostate cancer models, including cell lines, xenografts, primary tumors grown in an explant culture system, and organoids derived from men with CRPC. Activity of CDKI-73 in these diverse systems and disease subtypes strongly supports its potential as a new therapeutic for prostate cancer.

Our study demonstrates that CDKI-73 has a multipronged mode of action in prostate cancer cells. First, by blocking phosphorylation of Ser2-RNAPII, CDKI-73 inhibits transcription and reduces levels of key cancer-promoting factors such as MYC, pro-survival members of the BCL-2 family, and AR. Second, CDKI-73 also suppresses CDK9-catalyzed Ser81-AR, a post-translational modification that is important for its activity [6–9]. Third, beyond affecting the levels and phosphorylation of transcriptional regulators with central roles in prostate cancer growth and progression – AR, MYC, and BRD4 – CDKI-73 would blunt the oncogenic transcriptional programs activated by these factors via reducing pSer2-RNAPII. The pleiotropic effects of CDKI-73 likely have important ramifications for prostate cancer treatment: i) they may mitigate the ability of cancer cells to effectively develop resistance mechanisms; and ii) they would be expected to potentiate the activity of standard-of-care AR-targeted therapies. Both of these concepts should be tested in future studies.

Despite its potent effects on AR expression and activity, CDKI-73 exhibited comparable efficacy in AR-dependent and AR-negative/independent models of prostate cancer. This suggests that CDK9 inhibitors could be an effective therapeutic strategy irrespective of AR status, which is an exciting prospect given the lack of treatment options for AR-independent disease subtypes, such as NEPC, which exhibit enhanced plasticity and aggressive features [72]. In AR-independent contexts, we posit that effects on other CDK9 downstream targets are the dominant mechanism underlying CDKI-73’s anti-cancer activity. Indeed, MYC, MCL-1, and BCL-2 are important oncoproteins in both AR-driven and AR-independent prostate cancer [18, 73]. This further highlights the ability of CDK9 inhibitors to impinge on multiple oncogenic pathways, one of the most attractive features of this therapeutic strategy. Nevertheless, more work should be undertaken to delineate the precise mechanism of action of CDK9 inhibitors in AR-positive versus AR-independent tumors to accelerate their translation to the clinic.

Viewed collectively, the work undertaken to date exploring CDKI-73 as a clinical development candidate is very encouraging. CDKI-73 exhibits minimal toxicity in mice and against a variety of normal human cells (this study and [21–24]). Moreover, CDKI-73 has favorable pharmaceutical properties and oral bioavailability (F = 54–85%) [22], which will facilitate the delivery of the drug to patients. Importantly, a phase I trial of CDKI-73 in acute myeloid leukemia has recently been completed (CTR20190521) and the drug was well-tolerated and showed signs of activity. A phase 2 trial of CDKI-73 in patients with relapsed or refractory acute myeloid leukemia has commenced. These previous and ongoing studies will accelerate efforts to test CDKI-73 in patients with solid tumors.

Like other targeted therapies in cancer, CDK9 inhibition alone may not be sufficiently efficacious as single-agent therapy. Therefore, we sought to explore alternative rational drug combination strategies. BRD4 and CDK9 have complementary and intimately connected roles in enhancing oncogenic gene expression programs in a variety of cancer types, especially in the context of MYC-driven tumors [10]. In prostate cancer, these two factors have been shown to cooperate to amplify AR signaling in a low-androgen environment [8]. Importantly, enhanced CDK9 activity - manifested by increased pSer81-AR - is a mechanism of resistance to BET bromodomain inhibitors [74]; conversely, inhibition of CDK9 activity can lead to a BRD4-dependent increase in MYC expression and activity [68]. These observations have provided a strong rationale for combining CDK9 and BRD4 inhibitors, a concept that has shown promise in preclinical models of small-cell lung cancer, rhabdoid tumors and leukemia [67–69]. Herein, we extend this earlier work by showing significant synergy between CDKI-73 and the BRD4 inhibitor AZD5153 in prostate cancer cell lines and organoid models. Since both CDKI-73 and AZD5153 are already in human trials, a pathway to clinical translation is feasible.

A limitation of this study is that anti-cancer effects unrelated to CDK9 inhibition cannot be ruled out. While CDKI-73 is one of the most potent CDK9 inhibitors (Ki ~ 3 nM) identified and has high selectivity against non-CDK kinases, it also inhibits CDK1, CDK2 and CDK7 in biochemical kinase assays [56]. Indeed, we demonstrated loss of pSer5-RNAPII, a key readout of CDK7 activity, in LNCaP cells treated with CDKI-73. However, the knowledge that CDKI-73 is ~6–80-fold more active against CKD9 than these other CDKs in vitro [56] and the pharmacodynamic readouts evaluated in this study (i.e. inhibition of RNAPII pSer2 and AR pSer81 phosphorylation, reduced levels of MYC and anti-apoptotic proteins, impingement of BRD4 activity and an overall reduction in transcription) collectively support the concept that inhibition of CDK9 is the major mechanism by which CDKI-73 exerts anti-tumor activity in prostate cancer.

Overall, our study strongly supports the pursuit of CDK9 as a therapeutic target in prostate cancer, particularly in aggressive, therapy-resistant disease contexts. The orally bioavailable CDK9 inhibitor CDKI-73 has a multipronged mode of action, suppressing oncogenic transcriptional programs driven by AR, MYC, and BRD4 as well as suppressing critical anti-apoptotic pathways. These promising findings lend considerable weight to additional evaluation of this drug, potentially in clinical trials.

## Supplementary information

Supplementary Tables and Figures

Dataset S1

## Data Availability

RNA-seq and ChIP-seq data generated for this study are available from NCBI’s Gene Expression Omnibus (GSE255309).
