# Author Correction: CDK9 inhibition constrains multiple oncogenic transcriptional and epigenetic pathways in prostate cancer

**DOI:** 10.1038/s41416-024-02862-w

**Published:** 2024-10-24

**Authors:** Razia Rahman, Muhammed H. Rahaman, Adrienne R. Hanson, Nicholas Choo, Jianling Xie, Scott L. Townley, Raj Shrestha, Ramin Hassankhani, Saiful Islam, Susanne Ramm, Kaylene J. Simpson, Gail P. Risbridger, Giles Best, Margaret M. Centenera, Steven P. Balk, Ganessan Kichenadasse, Renea A. Taylor, Lisa M. Butler, Wayne D. Tilley, Simon J. Conn, Mitchell G. Lawrence, Shudong Wang, Luke A. Selth

**Affiliations:** 1 Flinders University, College of Medicine and Public Health, Flinders Health and Medical Research Institute, Bedford Park, SA Australia; 2 Drug Discovery and Development, Clinical and Health Sciences, University of South Australia, Adelaide, SA Australia; 3 Biomedicine Discovery Institute Cancer Program, Prostate Cancer Research Group, Department of Anatomy and Developmental Biology, Monash University, Clayton, VIC Australia; 4 Flinders University, Freemasons Centre for Male Health and Wellbeing, Bedford Park, SA Australia; 5 Victorian Centre for Functional Genomics, Peter MacCallum Cancer Centre, Melbourne, VIC Australia; 6 The Sir Peter MacCallum Department of Oncology, University of Melbourne, Parkville, VIC Australia; 7 Department of Biochemistry and Pharmacology, University of Melbourne, Parkville, VIC Australia; 8 Peter MacCallum Cancer Centre, Melbourne, VIC Australia; 9 Cabrini Institute, Cabrini Health, Malvern, Melbourne, VIC Australia; 10 Melbourne Urological Research Alliance (MURAL), Monash Biomedicine Discovery Institute Cancer Program, Monash University, Clayton, VIC Australia; 11 South Australian Health and Medical Research Institute, Adelaide, SA Australia; 12 Faculty of Health and Medical Sciences, The University of Adelaide, Adelaide, SA Australia; 13 Beth Israel Deaconess Medical Center, Boston, MA USA; 14 Department of Medical Oncology, Flinders Medical Centre, Southern Adelaide Local Health Network, Adelaide, SA South Australia; 15 Biomedicine Discovery Institute Cancer Program, Department of Physiology, Monash University, Clayton, VIC Australia; 16 Dame Roma Mitchell Cancer Research Laboratories, Adelaide Medical School, The University of Adelaide, Adelaide, SA Australia

**Keywords:** Targeted therapies, Prostate cancer, Oncogenes, Drug development

Correction to: *British Journal of Cancer* (2024) 131:1092-1105; 10.1038/s41416-024-02810-8, published online 08 August 2024

In this article the title was incorrectly given as “CDK9 inhibition inhibits multiple oncogenic transcriptional and epigenetic pathways in prostate cancer” but should have been “CDK9 inhibition constrains multiple oncogenic transcriptional and epigenetic pathways in prostate cancer”.

The original article has been corrected.

