# Supplementary material for: CDK9 inhibition constrains multiple oncogenic transcriptional and epigenetic pathways in prostate cancer
Source: Br J Cancer. 2024 Aug 8;131(6):1092–105. doi: 10.1038/s41416-024-02810-8 (PMC11405875; doi:10.1038/s41416-024-02810-8)
Supplement: Supplementary file 1 — Supplementary Tables and Figures [file 41416_2024_2810_MOESM1_ESM.pdf]

Supplementary Table 1. Primers used in this study.

**Name**	**Sequence**
GAPDH_F	TGCACCACCAACTGCTTAGC
GAPDH_R	GGCATGGACTGTGGTCATGAG
ACTB_F	GGCCAACCGCGAGAAGAT
ACTB_R	ATCACGATGCCAGTGGTACG
HPRT1_F	GTTATGGCGACCCGCAG
HPRT1_R	ACCCTTTCCAAATCCTCAGC
BCL2_F	ATGGGATCGTTGCCTTATGC
BCL2_R	CAGTCTACTTCCTCTGTGATGTTGT
MCL1_F	AACAAAGAGGCTGGGATGGG
MCL1_R	TGCCAAACCAGCTCCTACTC
XIAP_F	CCGGCCCAAGTGAAAAGGT
XIAP_R	TGATGTCTGCAGGTACACAAGT
c-Myc_F	GCCACGTCTCCACACATCAG
c-Myc_R	TCTTGGCAGCAGGATAGTCCTT
FKBP5_F	AAAAGGCCAAGGAGCACAAC
FKBP5_R	TTGAGGAGGGGCCGAGTTC
TMPRSS2_F	GACCAAGAACAATGACATTGCG
TMPRSS2_R	GTTCTGGCTGCAGCATCATG
AR-FL_F	CCTGGCTTCCGCAACTTACAC
AR-FL_R	GGACTTGTGCATGCGGTACTCA

Supplementary Table 2. Patient and tumor characteristics for samples used in patient-derived explant experiments.

**Patient ID**	**Primary Gleason grade**	**Secondary Gleason grade**	**PSA at diagnosis**	**Age at prostatectomy**	**Pathologic stage**
32425R	4	3	5.7	62.5	PT3B
32438R	4	4	10.0	71.4	PT3B
32436R	4	3	17.8	74.5	PT3A
32453LA	3	4	4.9	62.4	PT3A
32473R	3	4	5.4	63.2	PT3A
32464R	4	3	4.6	74.9	PT2C
32479R	3	4	3.4	56	PT3A
32396L	3	4	4.1	56.5	PT2C

A

Cell line	GI_{50} nM (MTT assay)
LNCaP	59 (±21)
C4-2B	40 (±1)
22Rv1	43(±9)
DU145	76 (±7)
PC3	55 (±6)
MRC-5	7391 ± 1146
WI-38	2238 ± 151

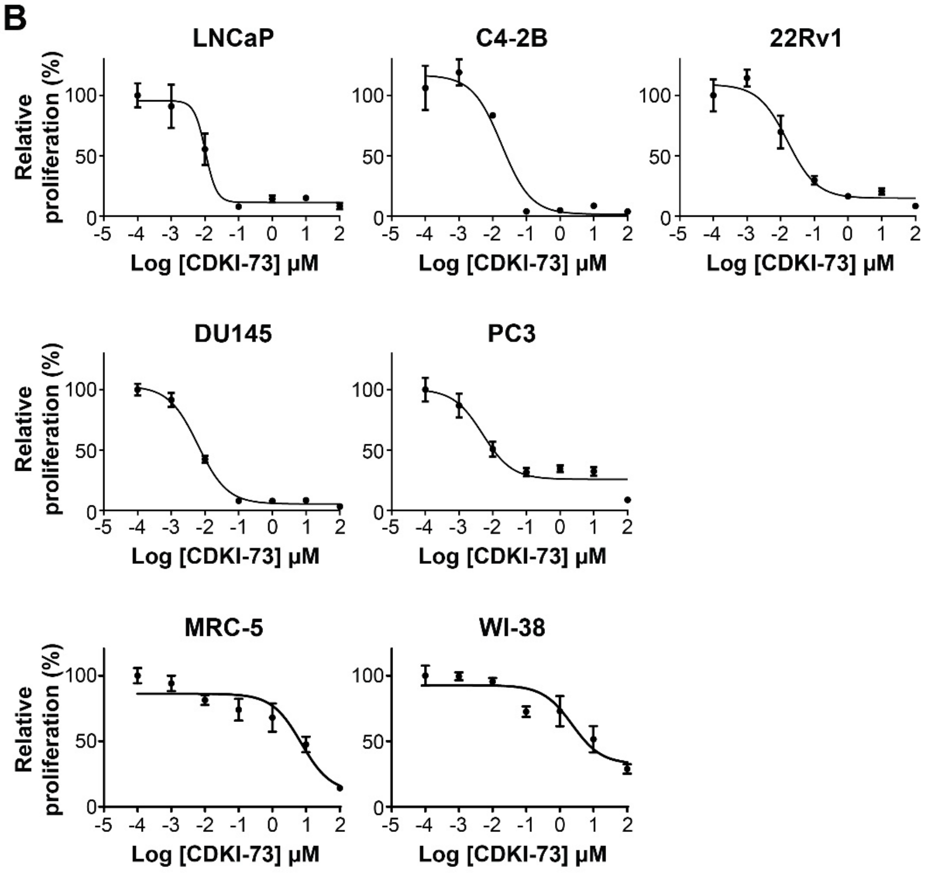

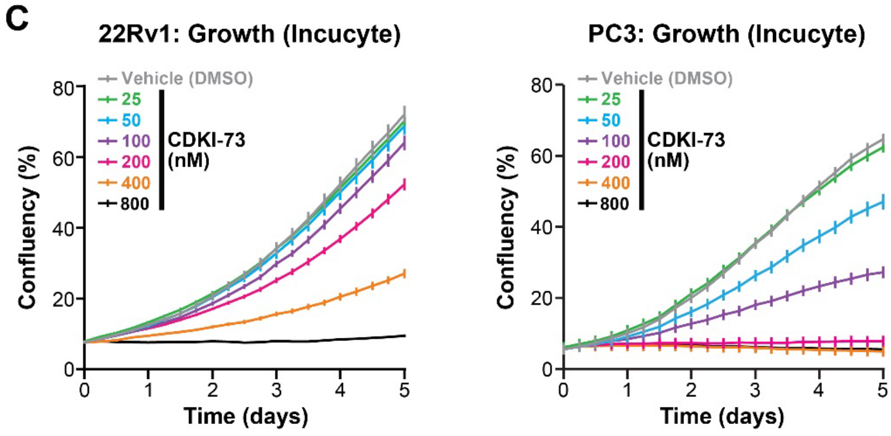

Supplementary Figure 1. ***In vitro*** **activity of CDKI-73 in prostate cancer cells. (A)** Half-maximal growth inhibitory concentration (GI_{50}) values (derived from MTT cell viability assays; see Materials and Methods) for CDKI-73 in 5 distinct prostate cancer cell line models and 2 non-malignant lung fibroblast cell lines (MRC-5 and WI-38). Standard error values are shown in brackets and are derived from 3 independent experiments. **(B)** Representative dose response curves from the MTT assay data shown in (A). **(C)** Live-cell confluency analysis (Incucyte) demonstrates a dose-dependent reduction of 22Rv1 and PC3 cell growth in response to CDKI-73. Error bars are ± SEM of 6 biological replicates. Data is representative of 3 independent experiments.

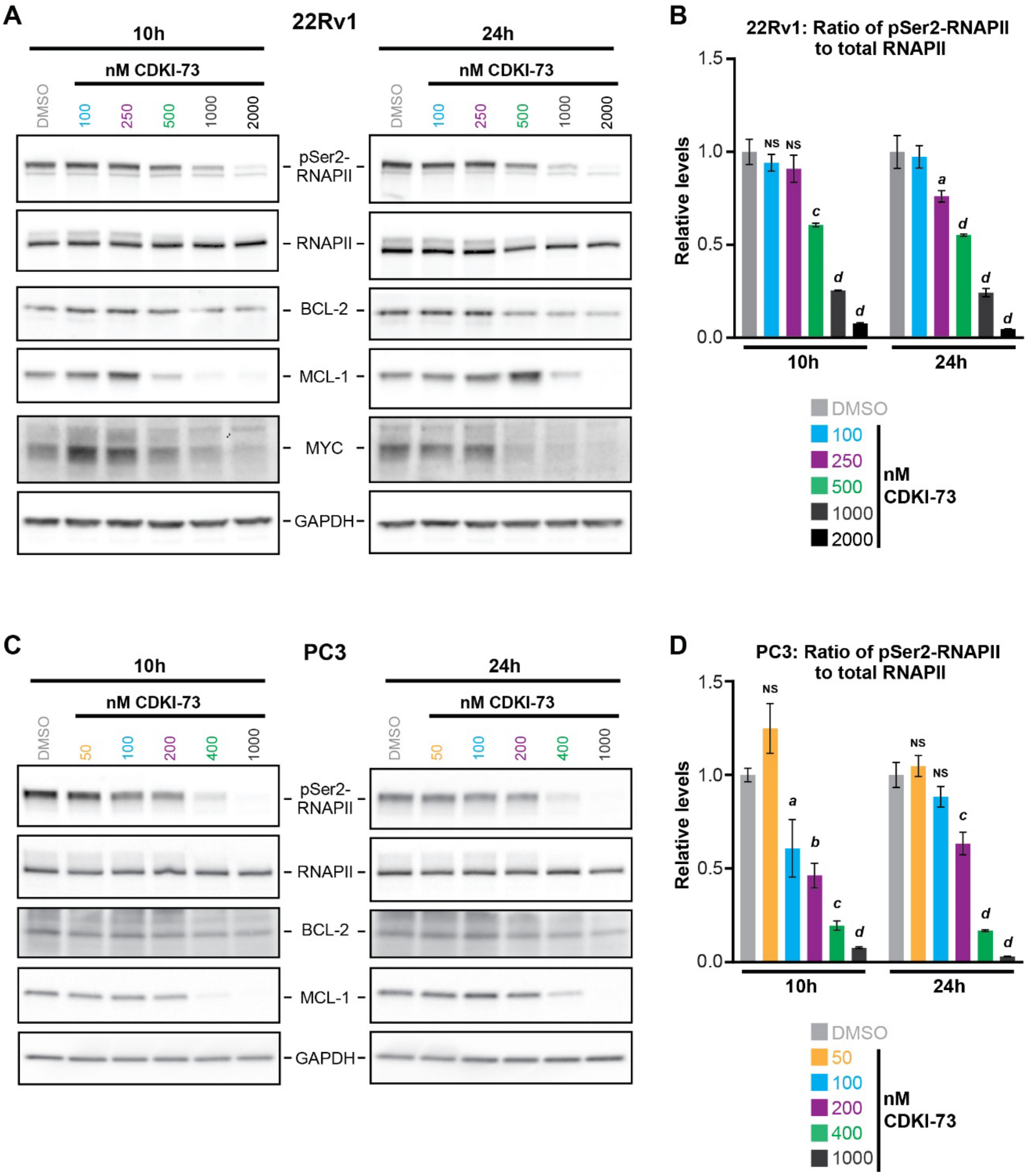

Supplementary Figure 2. CDKI-73 reduces the levels of pSer2-RNAPII and oncogenic proteins in prostate cancer cells. (A) Representative Western blots showing decreased levels of RNAPII, pSer2-RNAPII, BCL-2, MCL-1 and MYC following treatment of 22Rv1 cells with the indicated doses of CDKI-73 or vehicle control (DMSO) for 10 or 24 hours. GAPDH is shown as a loading control. **(B)** Normalized amount (ratio) of pSer2-RNAPII to total RNAPII (both normalized to GAPDH) following 10 and 24 hours of treatment with the indicated doses of CDKI-73 in 22Rv1 cells. DMSO was set to 1. Error bars are ± SEM of 3 biological replicates; P values (treatment compared to vehicle) were determined using ANOVA and Dunnett's multiple comparisons tests. In all panels: a, $p < 0.05$; b, $p < 0.01$; c, $p < 0.001$; d, $p < 0.0001$; NS, not significant. **(C)** Representative Western blots showing decreased levels of RNAPII, pSer2-RNAPII, BCL-2 and MCL-1 following treatment of PC3 cells with the indicated doses of CDKI-73 or vehicle control (DMSO) for 10 or 24 hours. GAPDH is shown as a loading control. **(D)** Normalized amount (ratio) of pSer2-RNAPII to total RNAPII (both normalized to GAPDH) following 10 and 24 hours of treatment with the indicated doses of CDKI-73 in PC3 cells. Data is presented and was analysed as in panel B.

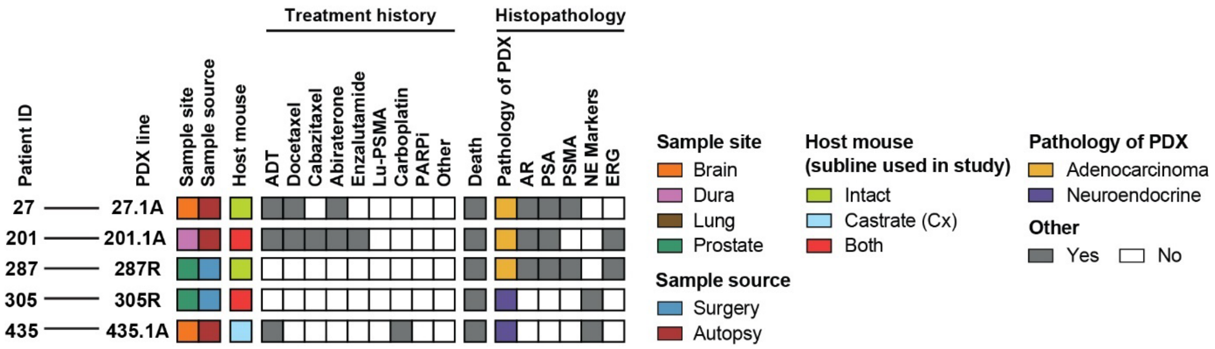

Supplementary Figure 3. Characteristics of prostate cancer PDX lines used in this study. The sample site, sample source, systemic therapies administered to patients prior to sample collection, clinical outcome at last follow-up, and pathology and biomarker expression of the PDXs are shown. Pathology of the PDXs was determined through histology review by pathologists and expression of phenotypic biomarkers by immunohistochemistry. NE marker staining indicates expression of ≥1 of chromogranin A, synaptophysin, and CD56. Cx denotes subline grown in castrated mice.

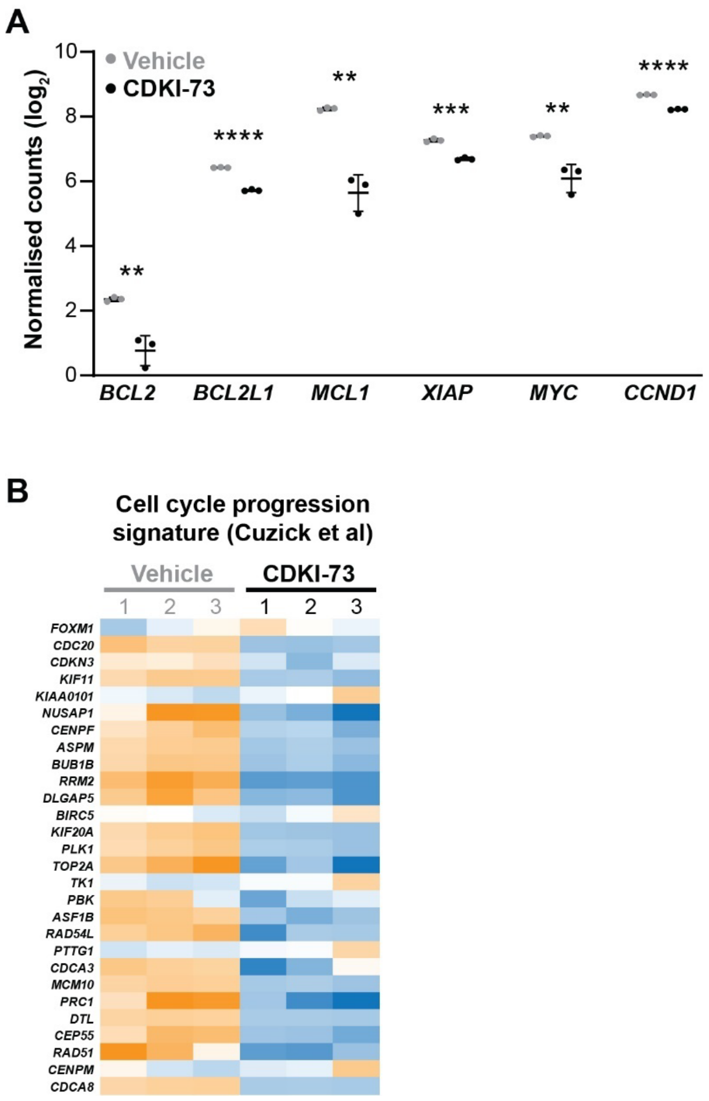

Supplementary Figure 4. Transcriptional effects of CDKI-73 in LNCaP prostate cancer cells. RNA sequencing demonstrates down-regulation of key CDK9-dependent target genes **(A)** and a cell cycle progression signature **(B)** by CDKI-73. In A, middle bars are the mean and top and bottom bars are ± standard deviation. P values (treatment compared to vehicle) were determined using t tests (**, $p < 0.01$; ***, $p < 0.001$; ****, $p < 0.0001$).

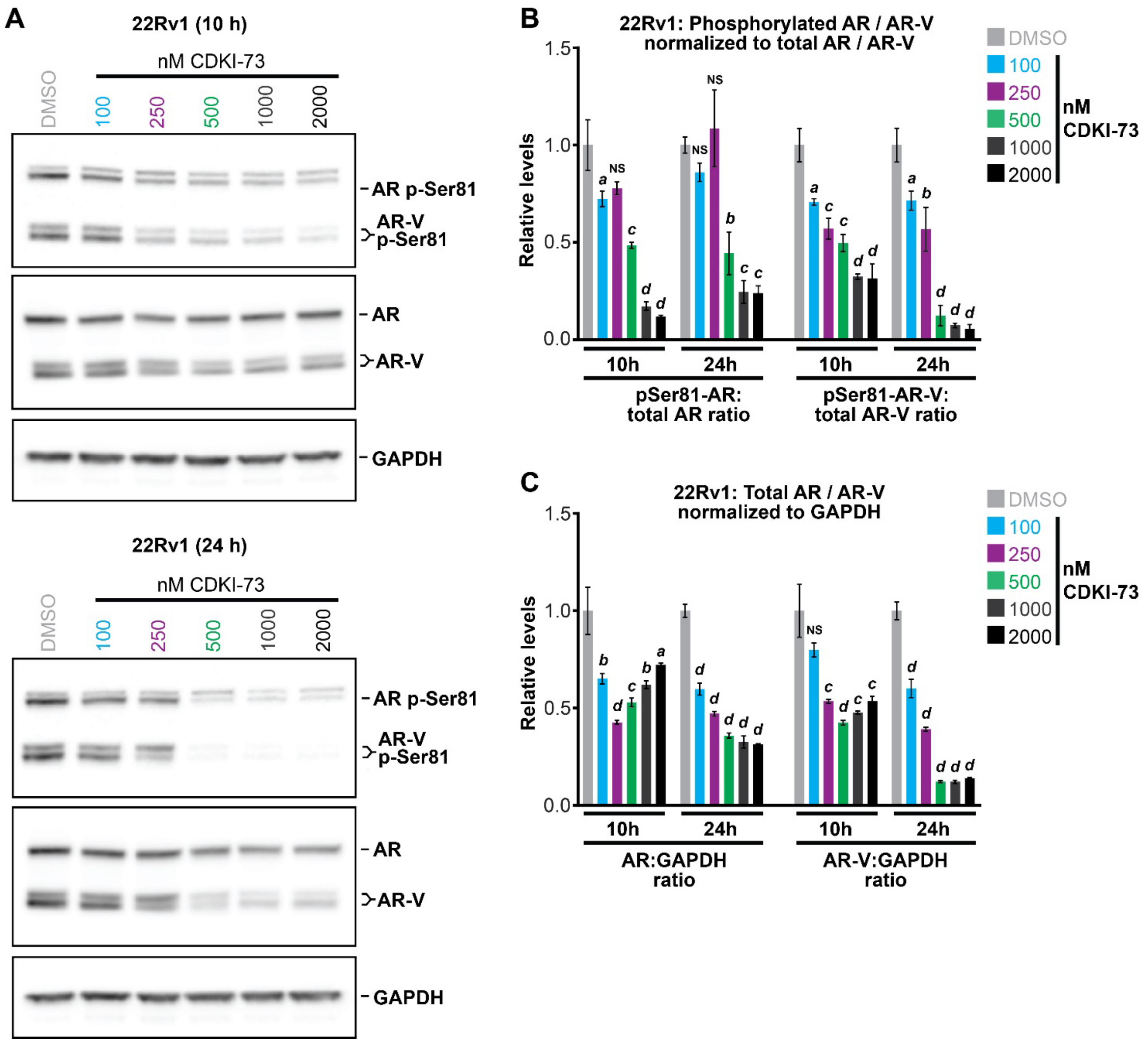

Supplementary Figure 5. CDKI-73 reduces the levels of AR and truncated AR variants in 22Rv1 cells. (A) Representative Western blots showing levels of pSer81-AR and total AR following treatment of 22Rv1 cells with the indicated doses of CDKI-73 or vehicle control (DMSO) for 10 and 24 hours. GAPDH is shown as a loading control. **(B)** Graph showing the normalized amount (ratio) of phosphorylated (pSer81) AR to total AR phosphorylated (pSer81) AR-Vs to total AR-Vs following 10 and 24 hours of treatment with the indicated doses of CDKI-73. All data was normalized to GAPDH and DMSO was set to 1. Error bars are ± SEM of 3 biological replicates; P values (treatment compared to vehicle) were determined using ANOVA and Dunnett's multiple comparisons tests. In all panels: a, $p < 0.05$; b, $p < 0.01$; c, $p < 0.001$; d, $p < 0.0001$; NS, not significant. **(C)** As for B, except data shows the amount of total AR or total AR-Vs (normalized to GAPDH) in response to CDKI-73.

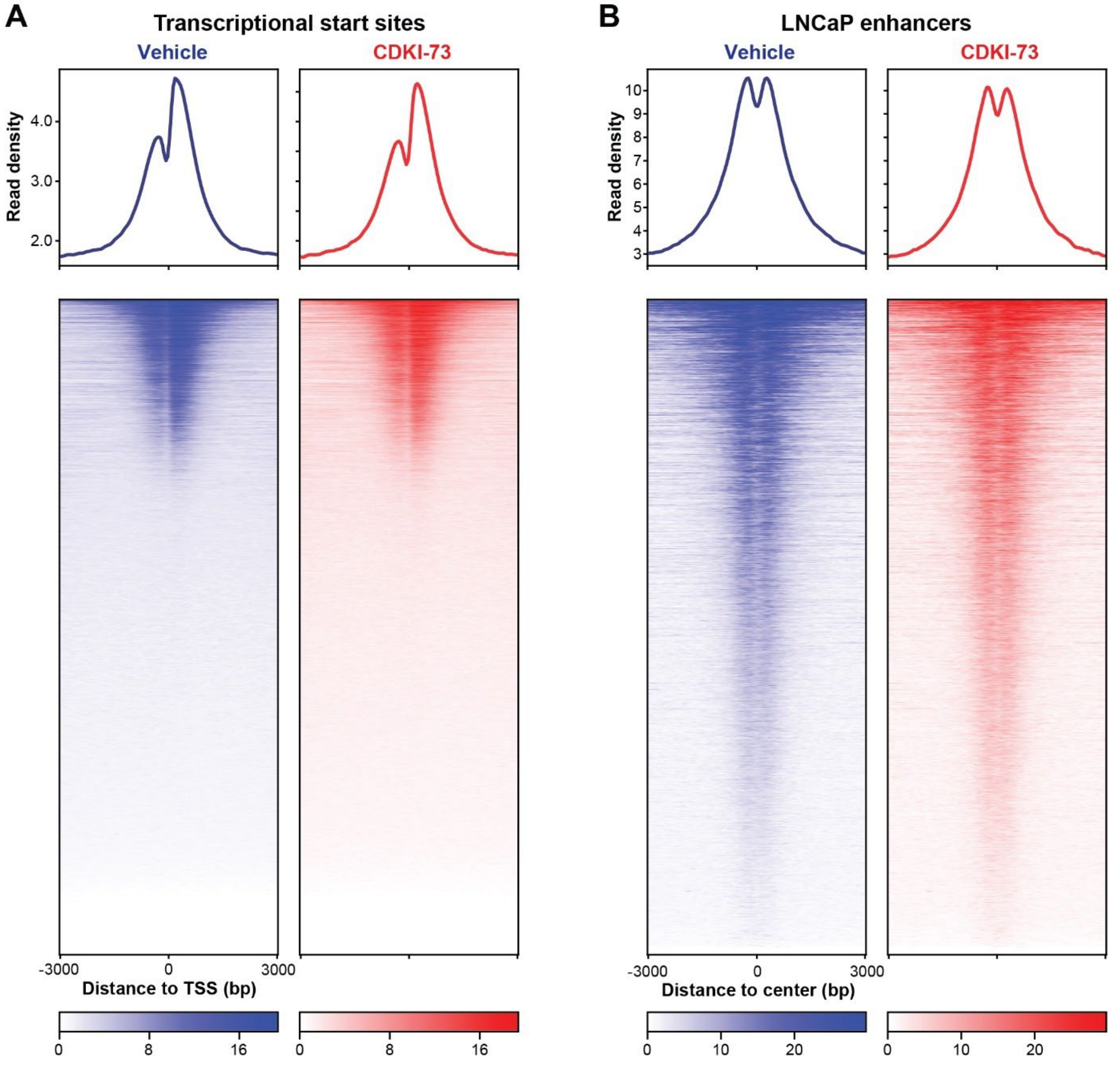

Supplementary Figure 6. Effect of CDKI-73 on H3K27ac at promoters and enhancers. Read density plots (top panels) and heatmaps (bottom panels) representing H3K27ac signal around transcriptional start sites **(A)** and gene enhancers **(B)**, the latter identified in LNCaP cells in a previous study (69).

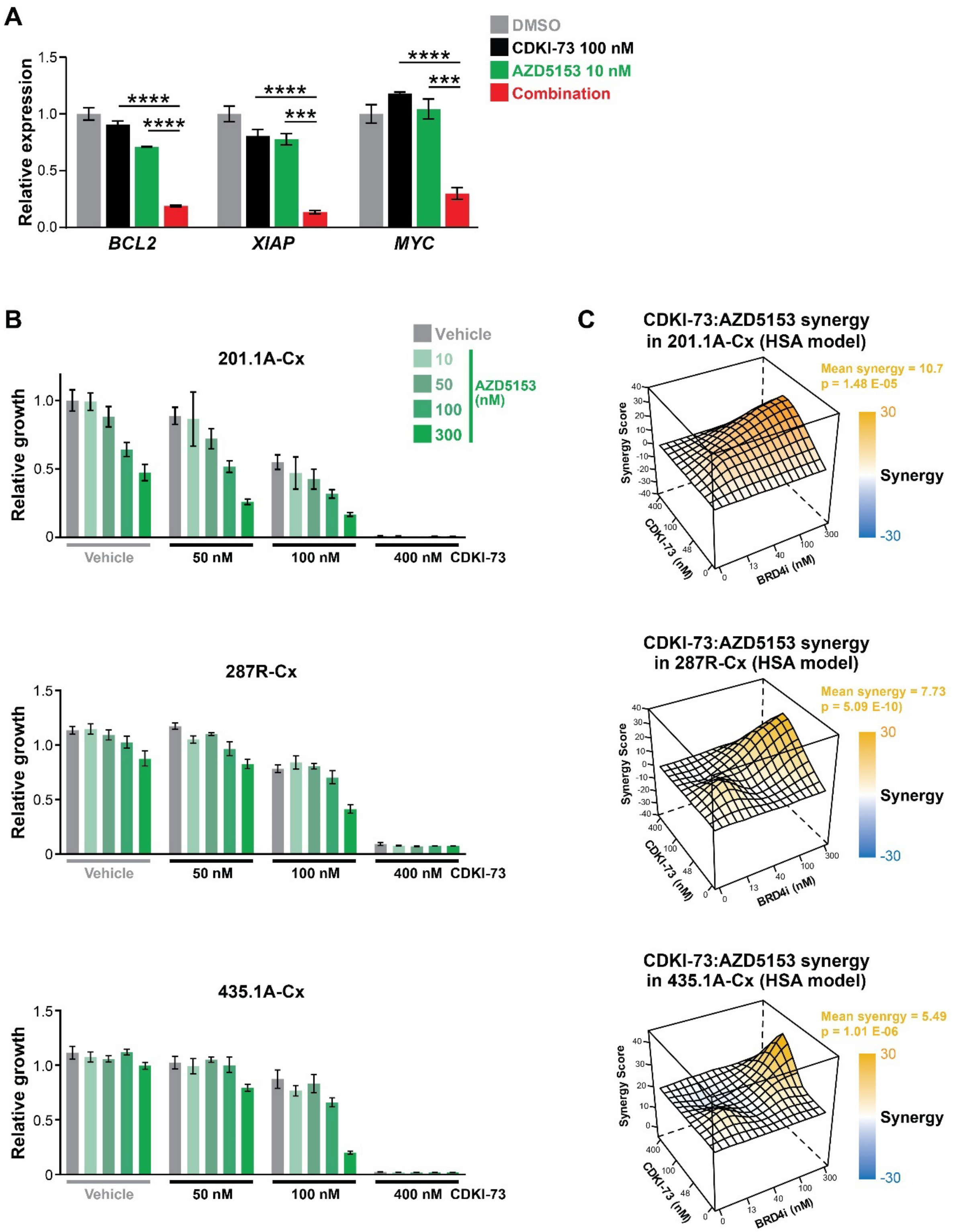

Supplementary Figure 7. Synergism of CDKI-73 with BRD4 inhibition. (A) Expression of *BCL2, XIAP* and *MYC* mRNA, as measured by qRT-PCR, in LNCaP cells following 8 hours of treatment with the indicated doses of CDKI-73, AZD5153 or the combination. Gene expression was normalized to *GAPDH* and *ACTB*; expression for vehicle (DMSO) was set to 1. Error bars are ± SEM of 3 biological replicates; P values (treatment compared to vehicle) were determined using ANOVA and Tukey's multiple comparisons tests. Data shown is representative of 2 independent experiments. **(B)** Combined activity of CDKI-73 and AZD5153 in organoid models. Organoid viability was determined using Cell Titer-Glo viability assays at 3 days post-treatment. Data represents the mean ± SEM of 6 individual wells. **(C)** Synergy map (highest single agent (HSA) model) for the experiments shown in A, generated using SynergyFinder Plus (see Materials and Methods).
